# The Recurrent Motor Branch of the Median Nerve

**Published:** 2013-12-24

**Authors:** Jeon Cha, Blair York, John Tawfik

**Affiliations:** The Sydney Hospital Hand Unit, Sydney Hospital and Sydney Eye Hospital, Sydney, Australia

**Keywords:** recurrent motor branch of the median nerve, median nerve, compressive neuropathy, carpal tunnel syndrome, non-operative management

**Figure:**
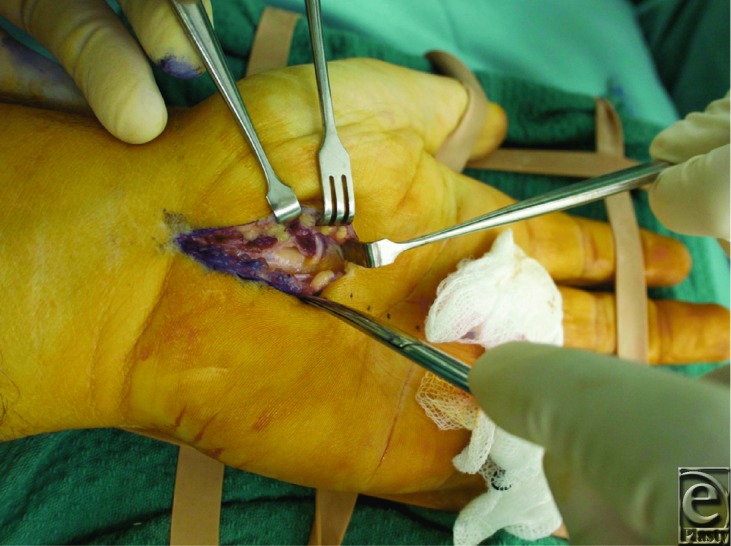


**Figure:**
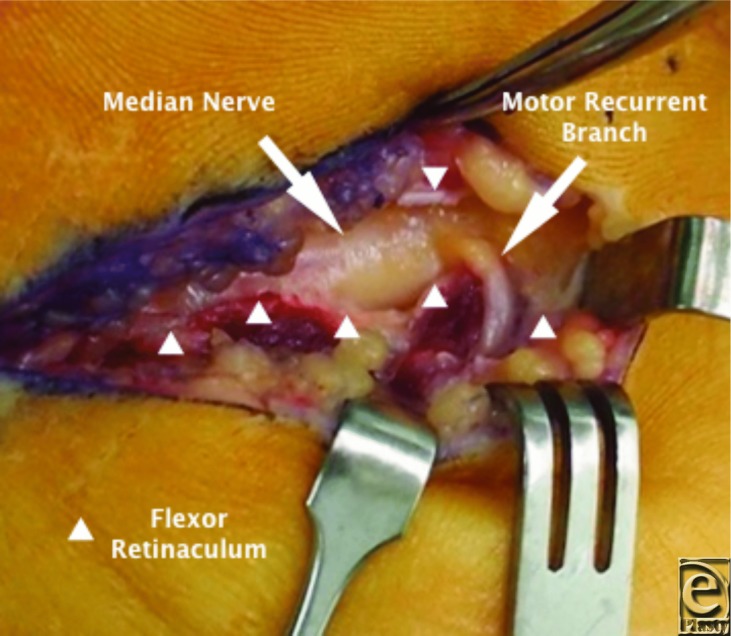


## DESCRIPTION

A 59-year-old man with bilateral carpal tunnel syndrome was treated surgically. During the release of the left side, a transligamentous recurrent motor branch was encountered. The patients' postoperative recovery was unremarkable with relief of nocturnal paresthesia and gradual improvement of motor function over the subsequent follow-up periods.

## QUESTIONS

1. **What are the symptoms and signs of carpal tunnel syndrome?**
2. **What are the causes of carpal tunnel syndrome?**
3. **What are the nonsurgical treatment options in carpal tunnel syndrome?**
4. **Discuss the variations of the recurrent motor branch.**

## DISCUSSION

Carpal tunnel syndrome is the most common compression neuropathy of the upper limb. Symptoms of carpal tunnel syndrome may include nocturnal pain/paresthesia involving the radial digits and increasing difficulty in fine motor tasks. Examination findings can include decreased sensation in the distribution of the median nerve (with a reduction in 2-point discrimination), preservation of cutaneous sensation to the thenar eminence, atrophy of the thenar musculature, and the ability to reproduce the symptoms and signs with provocative maneuvers (Phalen's, reverse Phalen's, Tinel's, Durkan's sign).^1^^,^^2^

Carpal tunnel syndrome results from increased pressure (≥32 mm Hg) within the carpal tunnel, leading to vascular ischemia of the median nerve.^3^ This pressure increase can result from factors that reduce the size of the tunnel, increase the volume of the contents, or secondary to underlying systemic conditions.^1^ A reduction in the size of the tunnel may be precipitated by mechanical/traumatic events such as volar carpal bone dislocation or volar migration of the base of the metacarpals. Volume increases from components within the carpal tunnel causing symptoms and signs are typically associated with anatomical variations. These include abnormal proximal lumbricals, low-riding flexor digitorum superficialis, a palmaris profundus, and a persistent median artery.^1^^,^^4^^,^^5^ Systemic conditions that can precipitate secondary carpal tunnel syndrome include gouty arthritis, rheumatoid arthritis, diabetes mellitus, hypothyroidism, and pregnancy.^1^

The treatment options for carpal tunnel syndrome are nonoperative and operative. Nonsurgical options that have demonstrated a clinical benefit in recent *Cochrane Reviews* include activity modification, steroid injections, nocturnal splinting, yoga, ultrasound therapy, and tendon gliding exercises.^6^^,^^7^

The most common course of the recurrent motor branch is extraligamentous distal to the flexor retinaculum. There are, however, significant variations in the course of the recurrent motor branch. Lanz^8^ in 1977 examined 246 hands in which carpal tunnel surgery was undertaken. Twenty-nine variations were identified that were classified into 4 groups. Variations involved the recurrent motor branch, those that were associated with accessory branches at the distal aspect of the carpal tunnel, anomalies with a high division of the median nerve, and those that were associated with accessory branches proximal to the carpal tunnel.^8^

Carpal tunnel syndrome is the most common compressive neuropathy that can be treated nonoperatively or surgically. If treated surgically, an awareness of the variations in the pattern of the recurrent branch of the median nerve is critical to avoid iatrogenic injury.
